# Development of depression in patients hospitalized for COVID-19 infection

**DOI:** 10.1192/j.eurpsy.2023.1657

**Published:** 2023-07-19

**Authors:** D. Lucijanić, A. Mihaljević-Peleš, N. Piskač-Živković, I. Rakoš, L. Mužinić Marinić

**Affiliations:** 1 Department of Psychiatry, University Hospital Dubrava; 2 Department of Psychiatry, University Hospital Center Zagreb; 3 Pulmonology, Special Hospital Radiochirurgia, Zagreb, Croatia

## Abstract

**Introduction:**

Coronavirus pandemic (COVID-19) has caused a great psychological impact all over the world. With this research, we want to discover the incidence and associated risk factors for depressive symptoms among hospitalized patients. The objective is to investigate patients with criteria of a severe clinical picture and expressed systemic inflammatory response to SARS-CoV-2 coronavirus infection and if they develop mental disorders- depression, measured by Depression, anxiety and stress scale- DASS-21 scale. With this research, we also calculate the index of the immune-inflammatory response SII and test the hypothesis that people with higher SII will develop mental disorders more often. Demographic variables, comorbidities, COVID-19 severity criteria, and the intensity of the organism’s inflammatory response have also been examined. Psychiatric questionnaires were for the first time applied directly to patients with coronavirus infection during hospitalization.

**Objectives:**

To identify possible risk factors for depression and to investigate the association between disease severity and the occurrence of psychopathology among COVID-19 hospitalized patients.

**Methods:**

The subjects are patients suffering from COVID-19, older than 18 years who were hospitalized in the respiratory center KB Dubrava. After an interview and informed consent, demographic data was taken and two psychological questionnaires had been applied. Variables: patient characteristics -demographic data, experience of vulnerability, information on whether they have been previously treated psychiatrically, symptoms of anxiety, depression, stress, somatic comorbidities Intensity of systemic inflammation Severity of COVID-19.

**Results:**

A total of 169 patients hospitalized were analyzed. The median age of the patients was 65. There were (62.1%) men and (37.9%) women. On admission, most patients had a severe (134, 79.3%) or critical (17, 10.1%) form of COVID-19. The median Charlson comorbidity index was 3 points. Arterial hypertension was present in 101 (59.8%), diabetes mellitus 42 (24.9%), hyperlipoproteinemia 30 (17.8%), obesity 61 (36.1%), malignant disease 17 (10.1%) patients. 11 (6.5%) smoked and 7 (4.1%) patients consumed alcohol. The median CRPa was 72.75 mg /L. Median SII was 1741. During hospitalization, the median DASS21 score for depression was 14, for anxiety 8, and for stress 6. Regarding depression, it was absent in 49 (29%), mild in 27 (16%), moderate in 47 (27.8%), severe in 18 (10.7%) and extremely severe in 28 (16.6%) patients during hospitalization.

**Conclusions:**

Patients with symptoms of depression during hospitalization felt statistically significantly more likely to be in danger of life due to COVID-19, had a more pronounced intensity of symptoms of COVID-19 upon admission. Additionally, patients with higher DASS 21 scores for depression were significantly more likely to be female, had COPD and required oxygen supplementation at higher flows.

**Disclosure of Interest:**

None Declared

